# Partially Virtual vs In-Person Care in Cardiovascular Conditions—Time Trends, Patient Characteristics, and Medication Management: Cohort Study

**DOI:** 10.2196/94061

**Published:** 2026-09-01

**Authors:** Sebastian Kinnberg Nielsen, Alessandra Meddis, Paul Frédéric Blanche, Mads Hashiba, Morten Schou, Ulrik Bak Kirk, Lars Kayser, Christian Torp-Pedersen, Nina Nouhravesh, Signe Stelling Risom, Anders Holt, Morten Kjøbek Lamberts

**Affiliations:** 1 Department of Cardiology, Copenhagen University Hospital – Herlev and Gentofte, Gentofte Hospitalsvej 6, postbox 635, Hellerup, Capital Region, DK-2900, Denmark, 45 40 47 95 56; 2 Section of Biostatistics, Department of Public Health, University of Copenhagen, Copenhagen, Capital Region, Denmark; 3 Department of Public Healh, Aarhus University, Aarhus, Central Jutland, Denmark; 4 Research Unit for General Practice, Aarhus University, Aarhus, Central Jutland, Denmark; 5 Department of Public Health, University of Copenhagen, Copenhagen, Capital Region, Denmark; 6 Department of Cardiology, Nordsjællands Hospital, Hilleroed, Capital Region, Denmark; 7 Institute of Nursing and Nutrition, University College Copenhagen, Copenhagen, Capital Region, Denmark; 8 Institute of Clinical Medicine, University of Copenhagen, Copenhagen, Capital Region, Denmark

**Keywords:** telemedicine, virtual care, cardiovascular disease, guideline-directed medical therapy, GDMT, follow-up, remote consultation, digital health, cardiology

## Abstract

**Background:**

Despite growing initiatives to promote partially virtual care, there is still a lack of comprehensive data describing its use, patient characteristics, and medication management practices.

**Objective:**

This study aimed to examine the use of partially virtual care in the management of cardiovascular disease.

**Methods:**

We used nationwide Danish registries to identify all patients with a first-time diagnosis of atrial fibrillation (AF), heart failure (HF), pulmonary embolism (PE), or acute coronary syndrome (ACS) between 2019 and 2023. Patients were categorized as receiving partially virtual care if followed up at least once virtually or in-person care if seen solely in-person. We evaluated temporal changes in the use of partially virtual care and calculated the odds of receiving partially virtual care vs in-person care and the prevalence of initiating guideline-directed medical therapy (GDMT). For GDMT initiation, the exposure was instead the type of the patient’s first follow-up visit. Multivariable logistic regression was used to estimate the odds of receiving partially virtual care, adjusted for frailty score, residence, and period of diagnosis. Separate multivariable logistic regression models were then used to estimate adjusted average differences in the prevalence of GDMT initiation between virtual and in-person follow-up among patients not already on the medication, accounting for frailty score, residence, and period of diagnosis.

**Results:**

In total, 45,919 patients with AF, 30,482 with HF, 12,451 with PE, and 12,435 with ACS were followed up. Among these, 27.3% (AF: 12,517/45,919), 51.7% (HF: 15,749/30,482), 47.5% (PE: 5918/12,451), and 37.2% (ACS: 4627/12,435) had partially virtual follow-up. The use of partially virtual follow-up increased during the COVID-19 pandemic and remained high afterward. In 2023, 17.4% (475/2731) to 35.3% (876/2481) of patients’ first follow-up visits were virtual, depending on the condition. Patient-specific factors significantly associated with partially virtual care were patients residing in cities for those with AF (odds ratio [OR] 1.27, 95% CI 1.21‐1.34), HF (OR 1.38, 95% CI 1.31‐1.46), and PE (OR 1.14, 95% CI 1.04‐1.24), but not for patients with ACS, and “high frailty” for those with AF (OR 1.63, 95% CI 1.48‐1.80) and PE (OR 1.53, 95% CI 1.29‐1.81). A large percentage of patients were on GDMT prior to the first follow-up visit. Differences in new initiation of GDMT were small in absolute terms across conditions, regardless of virtual or in-person follow-up; for example, for patients with HF, 2.23% initiated sodium-glucose cotransporter-2 inhibitors following a virtual visit, vs 4.47% after an in-person visit (prevalence difference −2.24%, 95% CI, −2.61 to −1.87).

**Conclusions:**

Among patients who received follow-up, a considerable proportion of patients with cardiovascular disease (from 12,517/45,919, 27.3% to 15,749/30,482, 51.7%) received partially virtual care. The odds of receiving partially virtual care varied significantly based on patient characteristics, particularly place of residence and frailty score. Finally, small absolute differences were found in the initiation of new GDMT.

## Introduction

Partially virtual care, including video and phone consultations, is increasingly requested by patients, physicians, and health systems and has expanded substantially in outpatient care since the COVID-19 pandemic [1,2]. The adoption of partially virtual cardiovascular care may improve access, continuity, and patient convenience and may support disease monitoring and timely treatment adjustment [3]. However, the extent to which virtual consultations can safely replace in-person follow-up in routine cardiovascular care remains uncertain [4,5].

Evidence for partially virtual care in cardiovascular disease (CVD) has grown but remains heterogeneous. A systematic review and meta-analysis of telemedicine for CVD management found favorable effects for some outcomes, particularly in heart failure (HF), but most of the included interventions combined remote consultations with additional components such as telemonitoring, structured telephone support, education, symptom tracking, or clinical decision support [2]. More recent evidence similarly suggests that home telemonitoring in HF can reduce all-cause mortality and HF hospitalization, but the intervention models vary considerably in intensity, technology, staffing, and escalation pathways [6,7]. In atrial fibrillation (AF), a recent cluster-randomized trial found that a telemedicine-based integrated care model improved adherence to guideline-based AF care and reduced cardiovascular events compared with usual care [8]. In acute coronary syndrome (ACS), the TELE-ACS (Remote Acute Assessment of Patients With High Cardiovascular Risk Post-Acute Coronary Syndrome) trial showed that a postdischarge telemedicine model incorporating remote diagnostic data and cardiologist assessment reduced readmissions, emergency department attendance, unplanned coronary revascularization, and symptoms [9]. By contrast, evidence for pulmonary embolism (PE) follow-up remains less developed; recent literature mainly supports structured follow-up, anticoagulation review, patient education, and surveillance for post-PE syndrome, while direct evidence on virtual follow-up as a substitute for in-person care is limited [10,11].

Much of the existing evidence comes from randomized trials of complex, resource-intensive interventions, rather than of virtual consultations as delivered in routine clinical practice [8,9]. Observational studies may better reflect real-world care, but are often small, disease-specific, or based on selected populations, limiting generalizability [12,13]. Consequently, there remains a need for large-scale population-based data describing how partially virtual follow-up is used after major cardiovascular diagnoses, which patients receive partially virtual rather than in-person care, and whether medication management differs by consultation modality.

Perhaps due to this lack of evidence, there are currently no established guidelines specifying which patients diagnosed with AF, HF, PE, or ACS should receive partially virtual, rather than in-person, hospital follow-up. Additionally, standardized national protocols for remote follow-up of these conditions are lacking.

The primary aim of this study was therefore to examine the use of partially virtual hospital follow-up care after diagnoses of major CVDs. The study also sought to identify patient factors associated with referral to partially virtual care and to assess medication management, particularly the initiation of guideline-directed medical therapy (GDMT), among patients initially followed up virtually compared with those followed up in-person.

## Methods

### Data Sources

We used Danish nationwide registers to perform a nationwide cohort study. The registers have previously been described in detail [14-18]. Each individual in Denmark has a unique personal identifier that enables linkage across all registers. Notably, information on visit type (virtual or in-person) has only been available since April 2019 (Multimedia Appendix 1).

### Population

Patients aged 18 years or older were included if they received a first-time diagnosis of AF, HF, PE, or ACS between April 2, 2019, and October 21, 2023, and were alive at discharge. The date of diagnosis was defined as either the hospital discharge date or the date of the outpatient visit.

### Population Characteristics

Population characteristics were considered as of the date of diagnosis. All characteristics are presented, stratified by diagnosis (Multimedia Appendix 1). Sex, median age, residence, education, income, frailty, medication, and comorbidities were reported. A complete list of the *International Classification of Diseases, 10th Revision* (ICD-10) and ATC (Anatomical Therapeutic Chemical) codes is provided in Table S1 in Multimedia Appendix 1.

### Follow-Up Visits

In the absence of a national protocol for remote follow-up, follow-up periods were defined based on clinical knowledge of each disease and its standard management. Patients with HF typically undergo up-titration of their medication within 2 to 3 months [19]. Patients with AF often have early follow-up to assess symptom burden and decide on a rhythm-control or rate-control strategy, including potential cardioversion. Furthermore, if an echocardiogram is necessary, it is often performed within 3 months. Patients with PE usually have a visit after 3 to 6 months to assess treatment duration and, potentially, undergo an echocardiogram to assess right ventricular dysfunction [20]. Patients with ACS have more variable follow-up, which may include risk factor modifications, medication adjustments, and an echocardiogram. Accordingly, patients diagnosed with AF or HF were followed for 90 days, while those with PE or ACS were followed for 180 days from the date of diagnosis.

During the follow-up period, we examined outpatient follow-up visits related to CVD, defined as visits lasting less than 12 hours [21]. Only visits after the date of diagnosis were considered. To explore common follow-up patterns, we examined the frequency and combinations of virtual and in-person visits for each condition, as well as the total number of follow-up visits. Patients were then categorized into 3 groups based on the type of follow-up care they received. For patients with AF, PE, or ACS, categorization was based on the first 2 follow-up visits; for HF, it was based on the first 4 follow-up visits. Patients were categorized as having received partially virtual care if at least one of their follow-up visits was virtual. Those seen exclusively in-person during the follow-up period were categorized as receiving “in-person care.” Patients with no follow-up at the hospital or for whom all visits had an unknown visit type (n=199) were excluded from the main analysis (Figure S1 in Multimedia Appendix 1).

### Medication Management

GDMT was assessed by visit type, both prior to and within 7 days after the patient’s first follow-up visit. This information was used to evaluate treatment changes following the visit. The primary aim was to describe the initiation of new medications by visit type (virtual vs in-person) and to describe medication use prior to the first follow-up visit. Here, exposure was the first follow-up visit (virtual vs in-person) after diagnosis, rather than the type of care (any virtual visit vs exclusively in-person). For patients with HF, the following medications were considered as part of GDMT: beta-blockers, angiotensin receptor/neprilysin inhibitor, loop diuretics, mineralocorticoid receptor antagonist, renin-angiotensin system inhibitor, and sodium glucose cotransporter 2 inhibitor. For patients with AF or PE, anticoagulants were included. For patients with ACS, lipid-lowering drugs and antiplatelets were included. The medication data are from filled prescriptions.

### Statistical Analysis

Population characteristics are shown by disease using median and IQRs, counts, and percentages. The proportion of patients seen virtually at their follow-up visits is reported by year and disease, along with the total number of patients seen annually for each disease. Additionally, the number and proportion of patients hospitalized during the follow-up period were calculated separately for those receiving partially virtual vs in-person care.

Multivariable logistic regression models were used to estimate the odds of receiving partially virtual care. We investigated whether specific patient characteristics differed between those who received partially virtual vs in-person follow-up. Variables included sex (male patients vs female patients), age group (<60, 60‐69, 70‐79, 80‐89, and ≥90), residence (city, rural, or town), ethnicity (Danish descent, immigrant, or descendant), frailty score (low, medium, or high), type of department responsible for the initial diagnosis (cardiology vs noncardiology), and type of hospital (specialized vs community hospital; see Multimedia Appendix 1 for definition of variables). To study the association between each of the variables above and the use of partially virtual care, we adjusted for frailty, residence, and period of diagnosis. The aim of the adjustment was to estimate the association within subgroups of patients similar with respect to the adjusted variables. The period of diagnosis was divided into 4-month intervals including pre-COVID-19, during COVID-19, and post-COVID-19 periods. The COVID-19 period was defined as March 2020 to June 2020, with pre-COVID being 3 months prior to this period and post-COVID period 1 month after this period. Patients with missing variables were excluded (complete-case analysis).

GDMT was described by visit type for each disease. Medication use was assessed first for drugs retrieved 180 days before the first follow-up visit, then for medication retrieved at the date of and up to 7 days after the first follow-up visit. Patients were considered newly initiated on a given medication if they had not retrieved it prior to the follow-up visit but did so within 7 days after it. For medication prior to the first follow-up visit, the analyses were stratified by period of diagnosis, with 2020 divided into a yes or no COVID-19 period. To estimate the prevalence of GDMT initiation among patients not already on the medication, we fit a multivariable logistic regression model with the visit type of the first follow-up visit as the exposure, adjusting for frailty score, residence, and period of diagnosis, including an interaction between visit type and period of diagnosis. Period of diagnosis was a categorical variable with one level per calendar year and a separate level for the COVID-19 period (March-June 2020), and the remainder of 2020 was treated as a distinct non-COVID level. We then applied marginal standardization to estimate the standardized prevalence of GDMT initiation for each visit type and the adjusted average differences between them. Patients with missing variables were excluded (complete-case analysis). To evaluate the potential impact on the health care system, we also calculated the proportion of patients who had an in-person visit within 2 weeks following their first follow-up visit. Data management and analyses were performed using R (version 4.2.1 for Windows; R Foundation for Statistical Computing [22]).

### Post Hoc Analysis

Fewer patients than anticipated received follow-up care. To explore factors associated with the absence of follow-up, we used the same multivariable logistic regression models to estimate the odds of not receiving any follow-up, based on population characteristics, as described above. Additionally, we report the number and proportion of hospitalizations during the follow-up period among patients who did and did not receive follow-up care. To gain further insight into the population not included in the main analysis, we also describe the characteristics of patients who died before follow-up, aiming to better understand which patients were excluded from follow-up assessments at the hospital.

To test the sensitivity of our definitions of partially virtual and in-person care, we explored factors associated with receiving purely virtual follow-up vs purely in-person follow-up, complementing the main analysis of the odds of receiving partially virtual care.

### Ethical Considerations

Approval to use the data sources for research purposes was granted by the data-responsible institution in the Capital Region of Denmark. In Denmark, register-based studies that are performed for the sole purpose of statistics and scientific research do not require ethics committee approval or patient consent, in accordance with the Danish Data Protection Act [23] and the General Data Protection Regulation (GDPR) [24]. However, the study is part of a project registered with the data-responsible institution in the Capital Region of Denmark (approval number P-2019‐537).

## Results

### Characteristics of the Population

We included a total of 91,639 patients with AF, 52,361 with HF, 22,084 with PE, and 32,446 with ACS, all diagnosed for the first time between April 2, 2019, and October 21, 2023 (Table 1).

The number of patients who had at least 1 follow-up visit was 45,919 (50.1%) for AF; 30,482 (58.2%) for HF; 12,451 (56.4%) for PE; and 12,435 (38.3%) for ACS. Among those with follow-up, the proportion receiving partially virtual care was 27.3% (12,517/45,919) for AF; 51.7% (15,749/30,482) for HF; 47.5% (5,918/12,451) for PE; and 37.2% (4,627/12,435) for ACS.

**Table 1.** Population characteristics.

Demographics	Atrial fibrillation (N=91,639)	Heart failure (n=52,361)	Pulmonary embolism (n=22,084)	Acute coronary syndrome (n=32,446)
Sex				
Female, n (%)	39,149 (42.7)	20,976 (40.0)	10,691 (48.4)	10,791 (33.3)
Male, n (%)	52,490 (57.3)	31,385 (59.9)	11,393 (51.6)	21,655 (66.7)
Age (y), median (IQR)	75 (67-82)	76 (65-83)	72 (61-79)	68 (58-77)
Residence, n (%)				
City	24,062 (26.3)	13,751 (26.3)	6234 (28.2)	8066 (24.9)
Rural	35,121 (38.3)	20,057 (38.3)	8125 (36.8)	12,819 (39.5)
Town	30,247 (33.0)	17,117 (32.7)	7000 (31.7)	10,770 (33.2)
Missing	2209 (2.4)	1436 (2.7)	725 (3.3)	791 (2.4)
Education, n (%)				
Elementary or high school	30,524 (33.3)	20,155 (38.5)	7180 (32.5)	10,258 (31.6)
Vocational education	37,430 (40.8)	21,009 (40.1)	9207 (41.7)	14,100 (43.5)
Higher education	21,803 (23.8)	9878 (18.9)	5241 (23.7)	7280 (22.4)
Missing	1882 (2.1)	1319 (2.5)	456 (2.1)	808 (2.5)
Income, n (%)				
Highest 25%	23,507 (25.7)	10,417 (19.9)	5735 (26.0)	9774 (30.1)
Second 25%	23,067 (25.2)	12,476 (23.8)	5625 (25.5)	8264 (25.5)
Third 25%	22,842 (24.9)	14,198 (27.1)	5393 (24.4)	7000 (21.6)
Lowest 25%	21,885 (23.9)	15,041 (28.7)	5260 (23.8)	7247 (22.3)
Missing	338 (0.4)	229 (0.4)	71 (0.3)	161 (0.5)
Frailty, n (%)				
Low	55,702 (60.8)	28,220 (53.9)	12,740 (57.7)	22,067 (68.0)
Medium	30,296 (33.1)	19,523 (37.3)	7742 (35.1)	9089 (28.0)
High	5641 (6.2)	4618 (8.8)	1602 (7.3)	1290 (3.9)
Medication, n (%)				
Antiplatelets	24,852 (27.1)	16,046 (30.6)	4426 (20.0)	6981 (21.5)
Beta blockers	31,954 (34.9)	21,963 (41.9)	3988 (18.1)	6079 (18.7)
Antidiabetic medication	12,152 (13.3)	9477 (18.1)	2458 (11.1)	4724 (14.6)
Direct oral anticoagulants	31,423 (34.3)	15,272 (29.2)	2598 (11.8)	2417 (7.4)
Other lipid-lowering drugs	1752 (1.9)	1197 (2.3)	274 (1.2)	654 (2.0)
Loop diuretics	16,278 (17.8)	18,567 (35.5)	3317 (15.0)	2909 (8.9)
Mineralocorticoid receptor antagonist	4757 (5.2)	4454 (8.5)	957 (4.3)	1058 (3.3)
NSAID^a^	9859 (10.8)	5103 (9.7)	3296 (14.9)	3908 (12.0)
Renin angiotensin system inhibitor	40,627 (44.3)	25,605 (48.9)	7519 (34.0)	11,944 (36.8)
SGLT2i^b^	2526 (2.8)	2518 (4.8)	458 (2.1)	999 (3.1)
Statins	34,853 (38.0)	21,905 (41.8)	5798 (26.3)	9709 (29.9)
Vitamin K antagonist	2967 (3.2)	3140 (5.9)	311 (1.4)	568 (1.8)
Comorbidities, n (%)				
Acute coronary syndrome	4433 (4.8)	8255 (15.8)	617 (2.8)	—^c^
Atrial fibrillation	—	18,407 (35.2)	1669 (7.6)	3204 (9.9)
Chronic kidney disease	4490 (4.9)	4137 (7.9)	931 (4.2)	1449 (4.5)
Chronic obstructive pulmonary disease	6550 (7.1)	6240 (11.9)	1948 (8.8)	1815 (5.6)
Heart failure	10,593 (11.6)	—	1330 (6.0)	3732 (11.5)
Pulmonary embolism	2128 (2.3)	1374 (2.6)	—	415 (1.3)
Stroke	7781 (8.5)	3519 (6.7)	1258 (5.7)	1252 (3.9)

a NSAID: nonsteroidal anti-inflammatory drug.

b SGLT2i: sodium glucose cotransporter 2 inhibitor.

c Index condition was excluded from the comorbidity lookback window to prevent self-referential counting.

### Follow-Up

#### Patterns of Follow-Up Visits

Absence of follow-up was more common than any combination of follow-up visits. Specifically, 49.9% (45,720/91,639) of patients with AF, 41.8% (21,879/52,361) with HF, 43.6% (9633/22,084) with PE, and 61.7% (20,019/32,446) with ACS had no follow-up visits. Among patients with AF, PE, and ACS, the most frequent follow-up pattern involved 1 or 2 in-person visits, followed by a combination of 1 in-person and 1 virtual follow-up visit. In contrast, patients with HF most commonly had between 1 and 4 in-person follow-up visits without any virtual visits. For patients who received partially virtual care, the typical pattern included 1 virtual visit combined with 1 to 3 in-person visits. The most common patterns of follow-up visits can be seen in Figure S2 in Multimedia Appendix 1. The majority of patients had a limited number of follow-up visits. No more than 2 follow-up visits during the follow-up period were recorded for 85.0% (77,918/91,639) of patients with AF, 79.0% (17,445/22,084) of patients with PE, and 82.4% (26,729/32,446) of patients with ACS. Among patients with HF, 81.4% (42,618/52,361) had a maximum of 4 follow-up visits.

#### Time Trends of Virtual Visits

**Figure 1.**
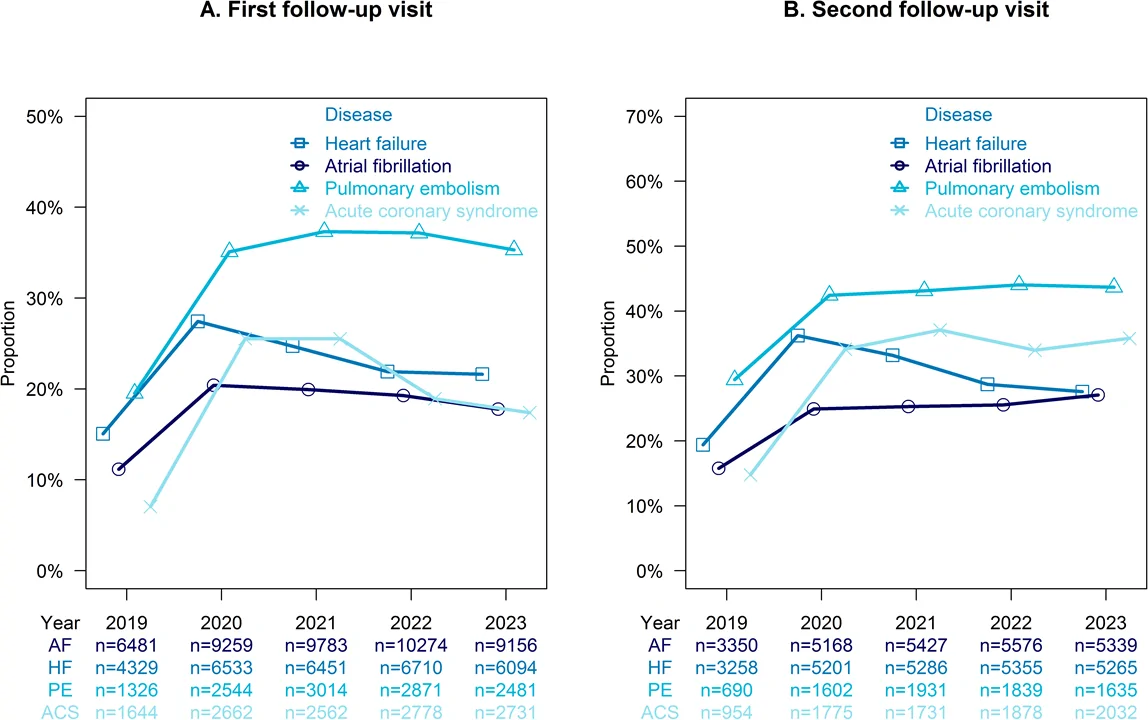
Proportion of virtual follow-up visits and total follow-up visits by year, stratified by disease. (A) First follow-up visit. (B) Second follow-up visit. ACS: acute coronary syndrome; AF: atrial fibrillation; HF: heart failure; PE: pulmonary embolism.

The use of virtual consultations increased between 2019 and 2020, peaking during the Danish national lockdown from March 2020 to June 2020 (Figure S3 in Multimedia Appendix 1) and has remained consistently high since. Among the 4 conditions studied, virtual consultations were most commonly used for patients with PE. By 2023, the proportion of virtual consultations for first and second follow-up visits was as follows: 17.8% (1627/9156) and 27.1% (1446/5339) for AF; 21.6% (1318/6094) and 27.6% (1452/5265) for HF; 35.3% (876/2481) and 43.7% (714/1635) for PE; and 17.4% (475/2731) and 35.8% (728/2032) for ACS (Figure 1).

#### Characteristics of Patients Receiving Partially Virtual Care

The odds of receiving partially virtual care varied significantly based on patient characteristics, particularly the place of residence, frailty score, and the type of hospital and department responsible for the initial diagnosis. Patients residing in cities had higher odds of receiving partially virtual care compared with those in rural settings. This difference was statistically significant for patients with AF (odds ratio [OR] 1.27, 95% CI 1.21‐1.34), HF (OR 1.38, 95% CI 1.31‐1.46), and PE (OR 1.14, 95% CI 1.04‐1.24).

**Figure 2.**
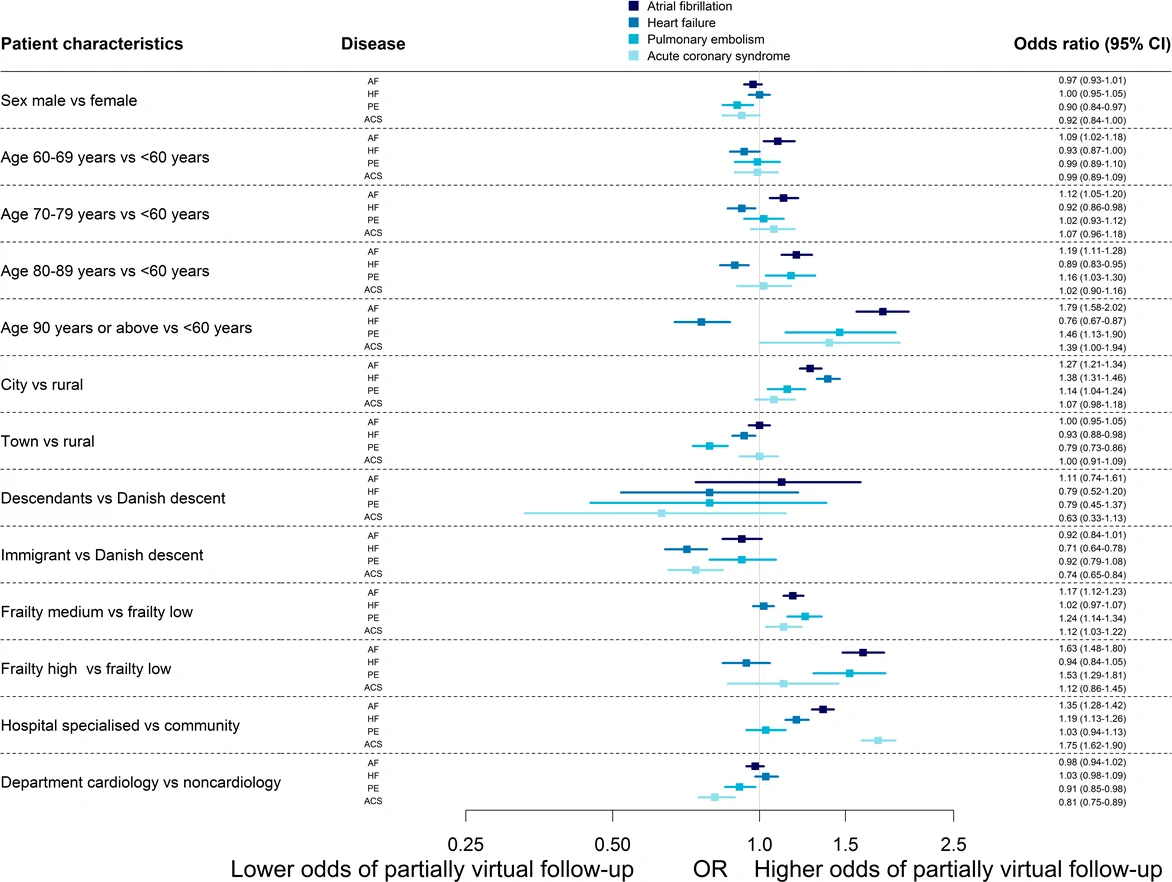
Forest plot of the odds of receiving partially virtual follow-up care by disease. ACS: acute coronary syndrome; AF: atrial fibrillation; HF: heart failure; OR: odds ratio; PE: pulmonary embolism.

A higher frailty score was associated with higher odds of receiving partially virtual care among patients with high frailty and AF (OR 1.63, 95% CI 1.48‐1.80) and among those with high frailty and PE (OR 1.53, 95% CI 1.29‐1.81). Patients diagnosed at specialized hospitals generally received partially virtual care more often than those diagnosed at community hospitals, except for patients with PE. However, a diagnosis at a cardiology department was associated with lower odds of partially virtual care among patients with PE (OR 0.91, 95% CI 0.85‐0.98) or ACS (OR 0.81, 95% CI 0.75‐0.89; Figure 2).

#### Medication Management

**Figure 3.**
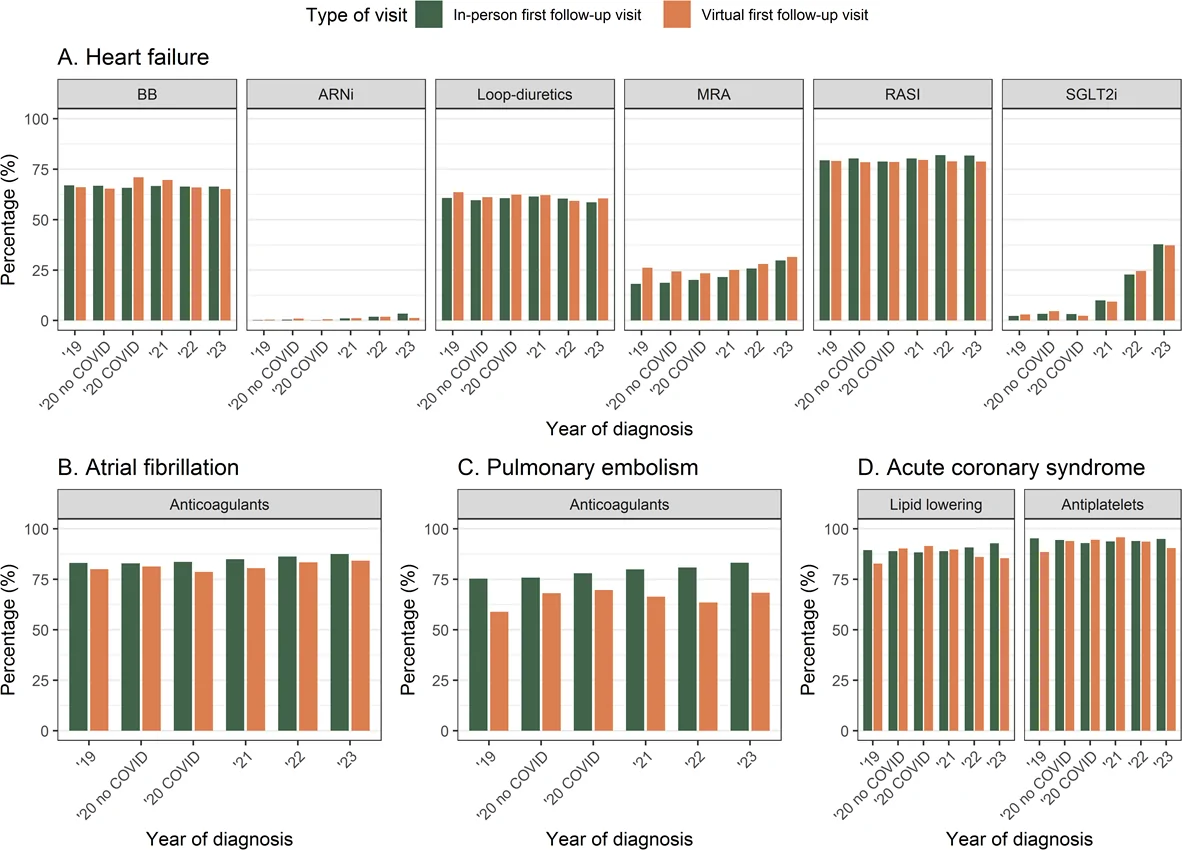
Guideline-directed medical therapy (GDMT) prior to the first follow-up visit, by disease: (A) heart failure, (B) atrial fibrillation, (C) pulmonary embolism, (D) acute coronary syndrome. ARNi: angiotensin receptor/neprilysin inhibitor; BB: beta-blockers; MRA: mineralocorticoid receptor antagonist; RASI: renin-angiotensin system inhibitor; SGLT2i: sodium-glucose cotransporter 2 inhibitor.

Most patients had already started GDMT before their first follow-up, irrespective of the visit type and disease. The uptake of sodium glucose cotransporter 2 inhibitors increased markedly from 2021 to 2023; otherwise, no major differences were found by year or during the national COVID-19 lockdown (Figure 3).

**Figure 4.**
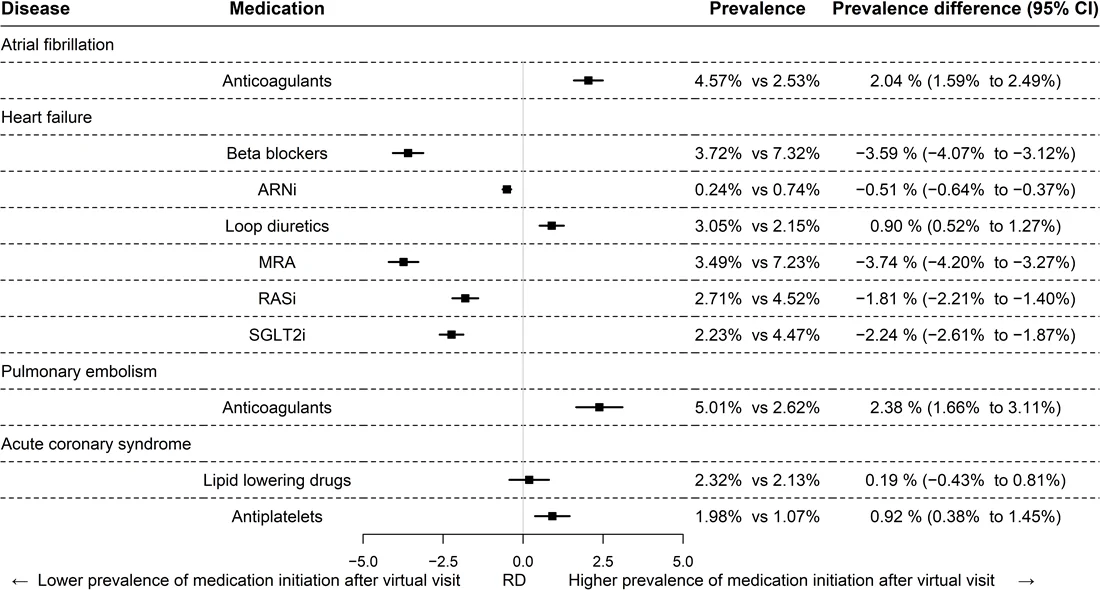
Prevalence difference in new treatment initiation after the first follow-up visit, virtual vs in-person. ARNi: angiotensin receptor/neprilysin inhibitor; MRA: mineralocorticoid receptor antagonist; RASI: renin-angiotensin system inhibitor; RD: risk difference; SGLT2i: sodium-glucose cotransporter 2 inhibitor.

Minor absolute differences were found in the initiation of GDMT following virtual vs in-person follow-up visits. For example, among patients with AF, an additional 4.57% of patients initiated anticoagulant treatment after a virtual visit compared with 2.53% after an in-person visit, yielding a prevalence difference of 2.04% (95% CI 1.59% to 2.49%; Figure 4).

### Reevaluations and Hospitalizations

Patients initially followed up virtually were generally more often seen again in-person within 14 days, compared with those whose first follow-up visit was in-person; for example, 28.5% (2326/8174) of those with AF initially seen virtually were seen again, while 20.3% (7454/36,789) of those seen in-person initially were seen again (Table S2 in Multimedia Appendix 1). Hospitalization occurred in 15.8% (1976/12,517; AF), 13.2% (2085/15,749; HF), 5.0% (298/5918; PE), and 6.5% (301/4627; ACS) of patients seen partially virtually, compared with 12.2% (4055/33,243; AF), 11.4% (1672/14,722; HF), 4.4% (289/6507; PE), and 7.3% (569/7805; ACS) of those seen in-person (Table S3 in Multimedia Appendix 1).

### Post Hoc Analysis

The odds of receiving follow-up care varied significantly based on patients’ frailty scores, residence, and the department responsible for the initial diagnosis. Patients with a high frailty score had lower odds of receiving follow-up compared with patients with a low frailty score. In general, patients residing in cities had higher odds of receiving follow-up care than those living in rural areas. Similarly, patients diagnosed in cardiology departments more often received follow-up, especially those with HF (OR 3.29, 95% CI 3.16‐3.41; Figure S4 in Multimedia Appendix 1). Hospitalization rates did not appear to be markedly higher among patients without follow-up. Among those who received follow-up, 13.2% (6075/45,919; AF), 12.3% (3760/30,482; HF), 4.8% (593/12,451; PE), and 6.9% (870/12,435; ACS) were hospitalized at least once, compared with 9.5% (4326/45,720; AF), 10.6% (2318/21,969; HF), 5.5% (528/9633; PE), and 5.1% (1029/20,011; ACS) among those without follow-up. Patients with AF or HF who died before follow-up were about 8 and 9 years older than survivors, while those with PE or ACS were around 6 and 14 years older (Table S4 in Multimedia Appendix 1).

For the odds of receiving purely virtual follow-up, we see similar trends in terms of patient characteristics associated with purely virtual follow-up, as we did with partially virtual care in the main analysis. For example, patients with AF (OR 1.33, 95% CI 1.23‐1.44), those with HF (OR 1.67, 95% CI 1.41‐1.99) and those residing in cities had higher odds of receiving purely virtual follow-up compared with patients living in rural areas (Figure S5 in Multimedia Appendix 1).

## Discussion

### Principal Findings

In this nationwide study examining the clinical use of partially virtual care among more than 100,000 patients with major cardiovascular conditions who were followed up at the hospital, we found that partially virtual care accounted for 27.3% (12,517/45,919) to 51.7% (15,749/30,482) of hospital outpatient care, depending on the condition. The most common pattern of partially virtual care was a single virtual visit. Key factors associated with receiving partially virtual care included residing in cities, higher frailty, and being diagnosed at a specialized hospital across nearly all conditions. Medication management, measured by the initiation of GDMT prior to and after patients’ first follow-up visit, was high across all conditions, regardless of whether the initial follow-up was conducted virtually or in-person, with only minor absolute differences found.

Partially virtual care was introduced rapidly during the COVID-19 pandemic [1,25], a trend also reflected in our findings, which show that its use nearly doubled from 2019 to 2020 and peaked from March 2020 to June 2020, when a majority of patients were seen virtually. This surge likely means that there was less of a difference between patients seen virtually and in-person during this period than overall across the study period. Due to the speed of this implementation, concerns persist [26,27]. A key point of concern is the inability to perform physical examinations during virtual consultations [28,29]. This concern seems valid considering that we found that a higher proportion of patients who initially had a virtual follow-up were seen again in-person within 14 days, compared with those who initially had in-person follow-up. This may reflect the need for certain procedures, such as echocardiography, that cannot be conducted virtually, necessitating timely in-person follow-up. Alternatively, this may reflect unnecessary duplication of care, potentially placing strain on health care resources.

### Policymakers and Patients

Our data offer valuable insights for policymakers seeking to understand how partially virtual care has been implemented in clinical practice. A key advantage of partially virtual care is its potential to reduce disparities in access to hospital care, particularly by improving care availability for patients in rural areas and reducing the need for travel [30]. However, we observed that patients with CVD in rural areas had lower odds of receiving partially virtual follow-up than those in cities, a finding echoed by research from general practice and other countries [31-33]. Similarly, community hospitals make less use of partially virtual care compared with specialized hospitals. Whether this lower use reflects an unmet opportunity to improve access or appropriate clinical decisions is beyond what our data can conclude. As broadband access is nearly universal in Denmark, it is unlikely to explain the lower use of partially virtual care in rural areas or community hospitals. Instead, these differences may reflect lower patient engagement in rural populations or more traditional care practices in community hospitals.

Interestingly, although this is beyond the primary scope of this study, a substantial proportion of patients did not receive any outpatient follow-up at the hospital. We think that their follow-up is often handled solely by their general practitioner, but we were unable to confirm this since we only have data on hospital contacts. In descriptive, unadjusted analyses, hospitalization proportions appeared comparable between patients with and without follow-up, meaning that the analysis did not indicate any obvious differences between the groups beyond what is captured by the presented population characteristics.

Notably, higher frailty, rural residence, and diagnosis at noncardiology departments were associated with an absence of follow-up care. These findings highlight an important area for future research.

### Clinicians

It is increasingly evident that partially virtual care is becoming an integral part of routine clinical practice, and every clinician will encounter situations where they must determine its appropriateness for individual patients. Overall, the current evidence tentatively supports the noninferiority of partially virtual care compared with in-person care [4,34,35]. In our study, medication management strategies after a virtual follow-up visit and an in-person visit were observed with similar retrieval of GDMT prior to patients’ first follow-up visits across care modalities. Initiation of new medication, among those not already on the medication, was low with minor differences in absolute numbers between types of follow-up. The low initiation rates are likely a result of the high proportion of patients already receiving treatment, resulting in clinically similar proportions of patients on GDMT after their first follow-up visits.

### Management of Patients With Cardiovascular Disease

CVDs vary significantly in their clinical characteristics and management requirements, which means that follow-up protocols differ across conditions, and not all are equally suited to partially virtual care. For example, patients with HF often undergo multiple consultations for titration of GDMT, many of which can be managed virtually. Nonetheless, an early in-person echocardiogram is essential to evaluate treatment response [19]. These disease-specific management needs are likely reflected in the observed variations in the use of partially virtual care across conditions and help explain the differing patterns of use. For instance, we observed especially high use for patients with PE, which might suggest that this condition is better suited for virtual management and often does not require in-person consultation except to assess right ventricular dysfunction. It may also simply be that there is a stronger tradition for virtual consultations among doctors managing patients with PE. Certain types of medication may also be easier to prescribe virtually, such as lipid-lowering drugs and anticoagulation, which do not affect hemodynamics, unlike drugs such as renin-angiotensin system inhibitors.

### Comparison With Prior Work

The marked increase in virtual consultations observed in our study during the COVID-19 pandemic is consistent with international reports demonstrating rapid adoption of telemedicine across cardiovascular care during periods of restricted in-person access, followed by stabilization at lower but sustained levels postpandemic [1,12,36]. Such sustained use suggests that partially virtual care has become integrated into routine outpatient management.

Our findings diverge from some previous studies regarding the characteristics of patients receiving partially virtual care. Observational studies from the United States have reported that older age and higher comorbidity burden are associated with lower odds of telemedicine use [13,36], potentially reflecting digital exclusion or barriers related to technology access and health literacy. In contrast, higher frailty scores in our cohort were associated with increased odds of partially virtual follow-up. Moreover, the association with age appeared disease-specific. Among patients with AF, older age was associated with higher odds of partially virtual care, whereas the opposite pattern was observed in patients with HF. These findings may suggest that, within the Danish health care context, partially virtual follow-up is being used pragmatically to reduce treatment burden and improve accessibility for selected frailer populations, rather than preferentially serving younger and healthier patients. The universal health care setting and lower financial barriers to specialist follow-up may partly explain differences from those reported in more insurance-dependent health care systems.

Geographic disparities in access persisted in our study. Patients living in less urbanized areas were less likely to receive partially virtual care, mirroring observations from Danish general practice [32] and studies from the United States [8]. This finding contrasts with the frequently proposed potential of telemedicine to improve health care access in geographically underserved populations [3,37], suggesting that structural factors such as digital infrastructure, organizational routines, or implementation practices may influence uptake beyond theoretical accessibility benefits.

The implications of partially virtual care for CVD management remain uncertain and appear to vary by condition and intervention type. Randomized trials and meta-analyses in HF have demonstrated that telemedicine interventions, particularly those combining remote monitoring, structured follow-up, and treatment optimization, can reduce hospitalization and mortality and improve uptake of GDMT [2,6,7,38,39]. Similarly, recent evidence in AF suggests telemedicine-based integrated care may improve adherence to guideline-based management and reduce cardiovascular events [8], while telemedicine-supported postdischarge care following ACS has been associated with fewer readmissions and emergency department visits [9]. However, these interventions are typically multifaceted and extend beyond virtual consultations alone. In contrast, observational studies have suggested that greater clinician telemedicine use may be associated with lower initiation of GDMT among patients with HF and reduced ejection fraction [40].

In our study, differences in medication management following virtual vs in-person follow-up were generally modest in absolute terms. This may indicate that partially virtual care, as implemented in routine clinical practice, neither substantially improves nor impairs the initiation of pharmacological treatment. Alternatively, it may reflect the selective allocation of virtual follow-up to patient groups with differing clinical complexity or treatment needs. Together, these findings underscore the importance of distinguishing between comprehensive telehealth programs and routine virtual consultations when evaluating effectiveness across cardiovascular conditions.

### Strengths and Limitations

First, we were limited by the information available in the registers and thereby did not know what happened or was discussed at each visit, nor the reasoning behind choosing the specific type of follow-up. As a result, we had to extrapolate certain aspects when interpreting the data, introducing a potential risk of misinterpretation. Second, the Danish health care system’s high level of digitization, combined with general differences between health care systems, means that caution is advised when generalizing our findings to other contexts. Third, physicians may select patients preferentially for partially virtual or in-person follow-up based on clinical severity markers, such as ejection fraction. Since these clinical data were unavailable, we could not examine this aspect. Fourth, there are inherent differences between video and phone consultations, but our data on this are too incomplete to use, so we cannot differentiate meaningfully.

Despite these limitations, we believe they are outweighed by the unselected and comprehensive nature of the data. The large-scale and real-world setting of this study offers a unique opportunity to evaluate how partially virtual care is actually being implemented in routine clinical practice and to examine its implications—constituting a major strength of this study. Randomized trials are often preferred for assessing causal effects, but for the evaluation of how virtual consultations are used in the clinic, observational data are necessary. Although beyond the scope of this paper, evaluating the effect of virtual consultations is also challenging in a clinical trial, as follow-up is not a fixed intervention. Patients may require multiple consultations and shift between virtual and in-person consultations depending on clinical need, leading to frequent protocol deviations and substantial crossover.

### Conclusions

Partially virtual care has become a widely used approach for follow-up in patients with CVD. The use of partially virtual care was different depending on patient characteristics, particularly place of residence, frailty score, and the type of hospital and department responsible for the initial diagnosis. Importantly, the difference in the prevalence of initiation of GDMT prior to and following patients’ first virtual and in-person follow-up visits was minor in absolute numbers. This means we found no obvious major differences in medication management.

## Supplementary material

10.2196/94061 Multimedia Appendix 1 Supplementary methods and additional tables and figures supporting the study, including disease and medication definitions, patient flow, hospitalizations during follow-up, follow-up visit patterns, virtual follow-up during the COVID-19 lockdown, and forest plots of follow-up odds by disease.

## Abbreviations

ACS: acute coronary syndrome
AF: atrial fibrillation
ATC: Anatomical Therapeutic Chemical
CVD: cardiovascular disease
GDMT: guideline-directed medical therapy
GDPR: General Data Protection Regulation
HF: heart failure
ICD-10: *International Classification of Diseases*, 10th Revision
OR: odds ratio
PE: pulmonary embolism
TELE-ACS: Remote Acute Assessment of Patients With High Cardiovascular Risk Post-Acute Coronary Syndrome
