# Supplementary material for: Partially Virtual vs In-Person Care in Cardiovascular Conditions—Time Trends, Patient Characteristics, and Medication Management: Cohort Study
Source: J Med Internet Res. 2026 Sep 1;28:e94061. doi: 10.2196/94061 (PMC13533318; doi:10.2196/94061)
Supplement: Supplementary file 1 [file jmir-v28-e94061-s001.docx]

**Supplementary material:**

Table of Contents

- Supplementary methods
- Supplementary Table 1 Definitions ICD-10 and ATC-codes
- Supplementary Table 2 Proportion of patients seen again within 14 days of first follow-up visit, by visit type and disease
- Supplementary Table 3 Proportion hospitalized at least once during follow-up, by visit type and disease
- Supplementary Table 4 Patients who died before follow-up
- Supplementary Figure 1 Flow chart of the categorization of the follow-up protocols
- Supplementary Figure 2 Common follow-up visit patterns after diagnosis, by disease
- Supplementary Figure 3 Proportion of virtual follow-up visits during the COVID-19 lockdown in 2020, by month
- Supplementary Figure 4 Forest plot odds of having follow-up by disease
- Supplementary Figure 5 Forest plot odds of having purely virtual follow-up by disease

**Supplementary methods**

*Data sources*

We used Danish nationwide registers, including the Danish National Patient Register (DNPR), the National Prescription Register, the Danish Civil Registration System, and the Danish Education Registers, all of which have been previously described in detail [1–5]. Each individual in Denmark has a unique personal identifier (CPR number) that enables linkage across all registers. This allows for detailed mapping of every patient’s hospital visits, including the exact dates and times of admission and discharge. Diagnosis information includes the ICD-10 code, diagnosis type (e.g., primary, secondary, or referral diagnosis), and the type of visit (virtual or in-person). Notably, information on visit type has been available only since April 2019. Prescription data have been available nationwide since 1995, including the date of drug retrieval and Anatomical Therapeutic Chemical (ATC) classification codes.

*Population characteristics*

Population characteristics were considered at diagnosis. All characteristics are presented stratified by diagnosis. Comorbidities were considered present if recorded within five years prior to diagnosis. Patients were considered to be concomitantly treated with the given medication if a prescription was claimed within 180 days prior to diagnosis. The following comorbidities were included: ACS, AF, chronic kidney disease, HF, PE and stroke. Medication categories included antiplatelet agents, beta-blockers, anti-diabetic medication, direct oral anticoagulants, other lipid lowering drugs, loop diuretics, mineralocorticoid receptor antagonist, non-steroidal anti-inflammatory drugs, renin-angiotensin system inhibitor, sodium-glucose cotransporter 2 inhibitors (SGLT2i), statins, and vitamin K antagonists. A complete list of ICD-10 and ATC-codes is provided in Supplementary Table 1.

Income was described as household-adjusted personal income based on income data from the 10 years prior to diagnosis and categorized into quartiles. Educational level was defined as the highest level ever attained and grouped into three categories: elementary or high school (ISCED 1–2), vocational education (ISCED 3), and higher education (ISCED 5؎8) [6]. Place of residence was categorized as city, rural, or town, according to the European Commission’s classification, which is based on population size and population density thresholds [7]. Ancestry was categorized as of Danish descent, immigrant, or descendant. A descendant is a person born in Denmark, neither of whose parents is both a Danish citizen and born in Denmark. Frailty was assessed using the validated Hospital Frailty Risk Score [8], categorizing patients into low (<5 points), medium (5–15 points), or high (>15 points) frailty groups. The hospitals where the initial diagnosis was made were classified either as specialized hospitals or community hospitals. The diagnosing departments were categorized as either cardiology or non-cardiology departments.

**References**

1.	Jensen VM, Rasmussen AW. Danish education registers. *Scand J Public Health*. 2011;39(7_suppl):91-94. doi:10.1177/1403494810394715

2.	Sundbøll J, Adelborg K, Munch T, et al. Positive predictive value of cardiovascular diagnoses in the Danish National Patient Registry: a validation study. Published online November 1, 2016. doi:10.1136/bmjopen-2016-012832

3.	Schmidt M, Pedersen L, Sørensen HT. The Danish Civil Registration System as a tool in epidemiology. *Eur J Epidemiol*. 2014;29(8):541-549. doi:10.1007/s10654-014-9930-3

4.	Schmidt M, Schmidt SAJ, Sandegaard JL, Ehrenstein V, Pedersen L, Sørensen HT. The Danish National Patient Registry: a review of content, data quality, and research potential. *Clin Epidemiol*. 2015;7:449-490. doi:10.2147/CLEP.S91125

5.	The Danish National Prescription Registry - Helle Wallach Kildemoes, Henrik Toft Sørensen, Jesper Hallas, 2011. Accessed April 8, 2025. https://journals.sagepub.com/doi/10.1177/1403494810394717?url_ver=Z39.88-2003&rfr_id=ori:rid:crossref.org&rfr_dat=cr_pub%20%200pubmed

6.	International Standard Classification of Education (ISCED). Accessed June 26, 2025. https://ec.europa.eu/eurostat/statistics-explained/index.php?title=International_Standard_Classification_of_Education_(ISCED)

7.	Information on data - Degree of urbanisation - Eurostat. Accessed May 1, 2025. https://ec.europa.eu/eurostat/web/degree-of-urbanisation/information-data

8.	Gilbert T, Neuburger J, Kraindler J, et al. Development and validation of a Hospital Frailty Risk Score focusing on older people in acute care settings using electronic hospital records: an observational study. *The Lancet*. 2018;391(10132):1775-1782. doi:10.1016/S0140-6736(18)30668-8

| **Supplementary Table 1 Definitions ICD-10 and ATC-codes** | | |
| --- | --- | --- |
| **Study population** | | |
| Atrial fibrillation | Defined from diagnosis codes | ICD: DI48 |
| Heart failure | Defined from diagnosis codes | ICD: DI50, DI42, DI110, DJ819 |
| Pulmonary embolism | Defined from diagnosis codes | ICD: DI26 |
| Acute coronary syndrome | Defined from diagnosis codes | ICD: DI21, DI200 |
| **Medication** | | |
| Vitamin K antagonists | Defined from ATC-codes | ATC: B01AA03, B01AA04 |
| Direct oral anticoagulants | Defined from ATC-codes | ATC: B01AE07, B01AF01, B01AF02, B01AF03 |
| Renin angiotensin system inhibitor | Defined from ATC-codes | ATC: C09 |
| Loop diuretics | Defined from ATC-codes | ATC: C03C, C03EB01, C03EB02 |
| Beta-blockers | Defined from ATC-codes | ATC: C07A, C07B, C07C, C07D, C07E, C07F |
| Mineralocorticoid receptor antagonist | Defined from ATC-codes | ATC: C03DA |
| Sodium glucose cotransporter 2 inhibitor | Defined from ATC-codes | ATC: A10BK |
| Angiotensin receptor neprilysin inhibitor | Defined from ATC-codes | ATC: C09DX04 |
| Antiplatelets | Defined from ATC-codes | ATC: B01AC06, B01AC04, B01AC22, B01AC24 |
| Anti diabetic medication | Defined from ATC-codes | ATC: A10 |
| Non-steroidal anti-inflammatory | Defined from ATC-codes | ATC: M01A |
| Other lipid lowering drugs | Defined from ATC-codes | ATC: C10AX |
| Statins | Defined from ATC-codes | ATC: C10AA |
| **Comorbidities** | | |
| Stroke | Defined from diagnosis codes | ICD: DI60-64 |
| Chronic kidney disease | Defined from diagnosis codes | ICD: DE102, DE112, DE142, DE131, DI132, DI150, DI151, DN03, DN05-DN08, DN110, DN14-DN19, DN26, DN27, DN280, DN391, DQ61 |
| Chronic obstructive pulmonary disease | Defined from diagnosis codes | ICD: DJ44 |
| ATC: Anatomical Therapeutic Chemical system. ICD-10: 10th revision of the International Classification of Diseases system. | | |

**Supplementary Table 2 Proportion of patients seen again within 14 days of first follow-up visit, by visit type and disease**

| **Disease** | **Type of visit** | **Proportion seen in-person (%)** | **Proportion seen virtually (%)** | **Proportion seen virtually and in-person (%)** |
| --- | --- | --- | --- | --- |
| Atrial fibrillation | | | | |
|  | Virtual | 28.5 | 15.1 | 4.2 |
|  | In-person | 20.3 | 6.0 | 1.9 |
|  | Difference | 8.2 (7.1 to 9.3) | 9.1 (8.3 to 9.9) | 2.3 (1.9 to 2.8) |
| Heart failure | | | | |
|  | Virtual | 30.6 | 23.6 | 7.0 |
|  | In-person | 22.2 | 11.9 | 2.9 |
|  | Difference | 8.4 (7.2 to 9.6) | 11.7 (10.6 to 12.8) | 4.1 (3.4 to 4.7) |
| Pulmonary embolism | | | | |
|  | Virtual | 10.7 | 16.6 | 2.3 |
|  | In-person | 10.4 | 8.0 | 1.4 |
|  | Difference | 0.3 (-0.9 to 1.4) | 8.6 (7.4 to 9.9) | 0.9 (0.4 to 1.5) |
| Acute coronary syndrome | | | | |
|  | Virtual | 15.5 | 10.0 | 2.2 |
|  | In-person | 15.6 | 6.9 | 1.3 |
|  | Difference | -0.1 (-1.7 to 1.5) | 3.1 (1.8 to 4.4) | 0.9 (0.3 to 1.5) |

**Supplementary Table 3 Proportion hospitalized at least once during follow-up, by visit type and disease**

| **Disease** | **Type of follow up** | **Proportion hospitalized (%)** |
| --- | --- | --- |
| Atrial fibrillation | | |
|  | Virtual | 15.79 |
|  | In-person | 12.20 |
|  | No follow up | 9.46 |
| Heart failure | | |
|  | Virtual | 13.24 |
|  | In-person | 11.36 |
|  | No follow up | 10.59 |
| Pulmonary embolism | | |
|  | Virtual | 5.04 |
|  | In-person | 4.44 |
|  | No follow up | 5.48 |
| Acute coronary syndrome | | |
|  | No follow up | 5.14 |
|  | Virtual | 6.51 |
|  | In-person | 7.29 |

| **Supplementary Table 4 Patients who died before follow-up** | | | | | | | | | |
| --- | --- | --- | --- | --- | --- | --- | --- | --- | --- |
|  |  | AF alive (n= 86 967) | AF died (n= 4 672) | HF alive (n= 48 534) | HF died (n= 3 827) | PE alive (n= 19 556) | PE died (n= 2 528) | ACS alive (n= 30 809) | ACS died (n= 1 637) |
| **Demographics** | | | | | | | | | |
| Male |  | 50,173 (57.7) | 2,317 (49.6) | 29,413 (60.6) | 1,972 (51.5) | 10,189 (52.1) | 1,204 (47.6) | 20,722 (67.3) | 933 (57.0) |
| Female |  | 36,794 (42.3) | 2,355 (50.4) | 19,121 (39.4) | 1,855 (48.5) | 9,367 (47.9) | 1,324 (52.4) | 10,087 (32.7) | 704 (43.0) |
| Median age (IQR) |  | 75 [66, 81] | 83 [77, 90] | 75 [65, 82] | 84 [77, 90] | 71 [60, 79] | 77 [69, 84] | 67 [57, 76] | 81 [74, 87] |
| **Residence** | | | | | | | | | |
|  | City | 23,037 (26.5) | 1,025 (21.9) | 12,947 (26.7) | 804 (21.0) | 5,699 (29.1) | 535 (21.2) | 7,722 (25.1) | 344 (21.0) |
|  | Rural | 33,489 (38.5) | 1,632 (34.9) | 18,749 (38.6) | 1,308 (34.2) | 7,192 (36.8) | 933 (36.9) | 12,227 (39.7) | 592 (36.2) |
|  | Town | 28,948 (33.3) | 1,299 (27.8) | 16,029 (33.0) | 1,088 (28.4) | 6,264 (32.0) | 736 (29.1) | 10,283 (33.4) | 487 (29.7) |
|  | Missing | 1,493 (1.7) | 716 (15.3) | 809 (1.7) | 627 (16.4) | 401 (2.1) | 324 (12.8) | 577 (1.9) | 214 (13.1) |
| **Education** | | | | | | | | | |
|  | Elementary- or high school | 28,275 (32.5) | 2,249 (48.1) | 18,272 (37.6) | 1,883 (49.2) | 6,183 (31.6) | 997 (39.4) | 13,502 (43.8) | 598 (36.5) |
|  | Vocational education | 35,809 (41.2) | 1,621 (34.7) | 19,686 (40.6) | 1,323 (34.6) | 4,793 (24.5) | 448 (17.7) | 7,035 (22.8) | 245 (15.0) |
|  | Higher education | 21,153 (24.3) | 650 (13.9) | 9,367 (19.3) | 511 (13.4) | 8,189 (41.9) | 1,018 (40.3) | 9,512 (30.9) | 746 (45.6) |
|  | Missing | 1,730 (2.0) | 152 (3.3) | 1,209 (2.5) | 110 (2.9) | 391 (2.0) | 65 (2.6) | 760 (2.5) | 48 (2.9) |
| **Income** | | | | | | | | | |
|  | Highest 25% | 23,058 (26.5) | 5-10% | 10,095 (20.8) | 5-10% | 5,310 (27.2) | 15-20% | 9,593 (31.1) | 10-15% |
|  | Second 25% | 22,127 (25.4) | 940 (20.1) | 11,705 (24.1) | 771 (20.1) | 5,037 (25.8) | 588 (23.3) | 7,932 (25.7) | 332 (20.3) |
|  | Third 25% | 21,292 (24.5) | 1,550 (33.2) | 12,942 (26.7) | 1,256 (32.8) | 4,636 (23.7) | 757 (29.9) | 6,453 (20.9) | 547 (33.4) |
|  | Lowest 25% | 20,156 (23.2) | 1,729 (37.0) | 13,565 (27.9) | 1,476 (38.6) | 4,505 (23.0) | 755 (29.9) | 6,672 (21.7) | 575 (35.1) |
|  | Missing | 334 (0.4) | <5% | 227 (0.5) | <5% | 68 (0.3) | <5% | 159 (0.5) | <5% |
| **Frailty** | | | | | | | | | |
|  | Low | 54,154 (62.3) | 1,548 (33.1) | 27,103 (55.8) | 1,117 (29.2) | 11,649 (59.6) | 1,091 (43.2) | 21,435 (69.6) | 632 (38.6) |
|  | Medium | 28,061 (32.3) | 2,235 (47.8) | 17,666 (36.4) | 1,857 (48.5) | 6,654 (34.0) | 1,088 (43.0) | 8,343 (27.1) | 746 (45.6) |
|  | High | 4,752 (5.5) | 889 (19.0) | 3,765 (7.8) | 853 (22.3) | 1,253 (6.4) | 349 (13.8) | 1,031 (3.3) | 259 (15.8) |
| **Medication** | | | | | | | | | |
| Antiplatelets |  | 23,210 (26.7) | 1,642 (35.1) | 14,857 (30.6) | 1,189 (31.1) | 3,739 (19.1) | 687 (27.2) | 6,416 (20.8) | 565 (34.5) |
| Beta blockers |  | 30,403 (35.0) | 1,551 (33.2) | 20,459 (42.2) | 1,504 (39.3) | 3,395 (17.4) | 593 (23.5) | 5,575 (18.1) | 504 (30.8) |
| Antidiabetic medication |  | 11,393 (13.1) | 759 (16.2) | 8,717 (18.0) | 760 (19.9) | 2,063 (10.5) | 395 (15.6) | 4,341 (14.1) | 383 (23.4) |
| Direct oral anticoagulants |  | 30,334 (34.9) | 1,089 (23.3) | 14,017 (28.9) | 1,255 (32.8) | 2,353 (12.0) | 245 (9.7) | 2,146 (7.0) | 271 (16.6) |
| Other lipid-lowering drugs |  | 1,694 (1.9) | 58 (1.2) | 1,152 (2.4) | 45 (1.2) | 253 (1.3) | 21 (0.8) | 624 (2.0) | 30 (1.8) |
| Loop diuretics |  | 14,675 (16.9) | 1,603 (34.3) | 16,620 (34.2) | 1,947 (50.9) | 2,652 (13.6) | 665 (26.3) | 2,392 (7.8) | 517 (31.6) |
| Mineralocorticoid receptor antagonist |  | 4,445 (5.1) | 312 (6.7) | 4,155 (8.6) | 299 (7.8) | 813 (4.2) | 144 (5.7) | 973 (3.2) | 85 (5.2) |
| NSAID |  | 9,453 (10.9) | 406 (8.7) | 4,835 (10.0) | 268 (7.0) | 2,940 (15.0) | 356 (14.1) | 3,753 (12.2) | 155 (9.5) |
| Renin angiotensin system inhibitor |  | 38,751 (44.6) | 1,876 (40.2) | 24,042 (49.5) | 1,563 (40.8) | 6,635 (33.9) | 884 (35.0) | 11,242 (36.5) | 702 (42.9) |
| SGLT2i |  | 2,427 (2.8) | 99 (2.1) | 2,415 (5.0) | 103 (2.7) | 398 (2.0) | 60 (2.4) | 948 (3.1) | 51 (3.1) |
| Statins |  | 33,369 (38.4) | 1,484 (31.8) | 20,569 (42.4) | 1,336 (34.9) | 5,056 (25.9) | 742 (29.4) | 9,126 (29.6) | 583 (35.6) |
| Vitamin K antagonist |  | 2,828 (3.3) | 139 (3.0) | 2,896 (6.0) | 244 (6.4) | 280 (1.4) | 31 (1.2) | 511 (1.7) | 57 (3.5) |
| **Comorbidities** | | | | | | | | | |
| Acute coronary syndrome |  | 4,162 (4.8) | 271 (5.8) | 7,818 (16.1) | 437 (11.4) | 519 (2.7) | 98 (3.9) | – | – |
| Atrial fibrillation |  | – | – | 16,826 (34.7) | 1,581 (41.3) | 1,335 (6.8) | 334 (13.2) | 2,835 (9.2) | 369 (22.5) |
| Chronic kidney disease |  | 3,964 (4.6) | 526 (11.3) | 3,579 (7.4) | 558 (14.6) | 772 (3.9) | 159 (6.3) | 1,232 (4.0) | 217 (13.3) |
| Chronic obstructive pulmonary disease |  | 5,751 (6.6) | 799 (17.1) | 5,454 (11.2) | 786 (20.5) | 1,594 (8.2) | 354 (14.0) | 1,565 (5.1) | 250 (15.3) |
| Heart failure |  | 9,651 (11.1) | 942 (20.2) | – | – | 1,091 (5.6) | 239 (9.5) | 3,307 (10.7) | 425 (26.0) |
| Pulmonary embolism |  | 1,896 (2.2) | 232 (5.0) | 1,218 (2.5) | 156 (4.1) | – | – | 359 (1.2) | 56 (3.4) |
| Stroke |  | 7,116 (8.2) | 665 (14.2) | 3,088 (6.4) | 431 (11.3) | 1,040 (5.3) | 218 (8.6) | 1,088 (3.5) | 164 (10.0) |
| **Abbreviations:** *NSAID=* Non-steroidal anti-inflammatory drug, *IQR=* Interquartile range, *SGLT2i* =Sodium glucose cotransporter 2 inhibitor, – =The index condition was excluded from the comorbidity lookback window to prevent self-referential counting, percentage bands were used for the income variable to ensure anonymity. | | | | | | | | | |

Supplementary Figure 1: Flow chart of the categorization of the follow-up protocols

Legend: If at any time after initial diagnosis a patient had a virtual consultation during the follow-up period, they were considered as having received partially virtual follow-up. n: denotes the total number of follow-up visits for each disease. So n=2 for AF, PE and ACS and 4 for HF.

Supplementary Figure 2 Common follow-up visit patterns after diagnosis, by disease

Supplementary Figure 3 Proportion of virtual follow-up visits during the COVID-19 lockdown in 2020, by month

Legend: AF= Atrial fibrillation, HF= Heart failure, PE = Pulmonary embolism

 Supplementary Figure 4 Forest plot odds of having follow-up by disease

Legend: Multivariable logistic regression models were implemented to estimate the odds of receiving virtual care. Adjusted for frailty score, residence and period of diagnosis. AF= Atrial fibrillation, HF= Heart failure, PE = Pulmonary embolism, n = number of, OR = Odds ratio, CI= Confidence interval

 Supplementary Figure 5 Forest plot odds of having purely virtual follow-up by disease

Legend: Multivariable logistic regression models were implemented to estimate the odds of receiving virtual care. Adjusted for frailty score, residence and period of diagnosis. AF= Atrial fibrillation, HF= Heart failure, PE = Pulmonary embolism, n = number of, OR = Odds ratio, CI= Confidence interval
